# Neuroprotective Effects of Savinin on LPS-Induced Neuroinflammation In Vivo via Regulating MAPK/NF-κB Pathway and NLRP3 Inflammasome Activation

**DOI:** 10.3390/molecules28041575

**Published:** 2023-02-07

**Authors:** Siqi Tang, Chunying Li, Zongwu Suo, Yi Xu, Kaixin Wei, Lei Zhao, Hao Huang, Xiangqian Liu, Dongxu Liu, Xiaojun Li

**Affiliations:** 1 National Engineering Research Center for Modernization of Traditional Chinese Medicine—Hakka Medical Resources Branch, School of Pharmacy, Gannan Medical University, Ganzhou 341000, China; 2 College of Basic Medicine, Gannan Medical University, Ganzhou 341000, China; 3 School of Pharmacy, Hunan University of Chinese Medicine, Changsha 410208, China

**Keywords:** savinin, neuroinflammation, MAPK, NF-κB, NLRP3 inflammasome

## Abstract

The traditional herb *Eleutherococcus henryi* Oliv. is commonly used to treat inflammatory conditions including rheumatism, arthritis, and hepatitis, as well as mental fatigue and amnesia, according to traditional Chinese medicine (TCM) theory. Savinin is a natural lignan obtained from the roots of *E. henryi*. The present study was undertaken to determine whether savinin can relieve LPS-induced neuroinflammation and if so, what the mechanism is. Groups of male C57BL/6 mice were administered savinin (5, 10, 20 mg/kg) and DEX (10 mg/kg) by gavage once daily for a continuous 7 days. On the 5th day of continuous pre-administration, LPS (2.5 mg/kg) was injected into the lateral ventricles of the mice for modeling 48 h. We found that treatment with savinin decreased the levels of neuroinflammatory cytokines and histopathological alterations dramatically. Consequently, it improved the LPS-induced neuroinflammatory response in mice. Furthermore, savinin inhibited the up-regulated expression of related proteins in the activated MAPK/NF-κB and NLRP3 inflammasome signaling pathways caused by LPS. Docking studies demonstrated the binding of savinin to three receptors (MAPK, NF-κB and NLRP3) using a well-fitting mode. These findings suggest that savinin may suppress neuroinflammation induced by LPS in vivo via modulating MAPK/NF-κB and NLRP3 signaling pathways.

## 1. Introduction

Neuroinflammation refers to the self-protection mechanism initiated by the central nervous system (CNS) when abnormalities occur in the brain. It mainly removes the fragments of damaged cells through immune cells in the brain and regulates the secretion of neurotrophic factors to maintain brain homeostasis and normal physiological function [1]. However, the gradual formation of chronic neuritis shows neurotoxicity due to the large release of cytokines, and the excessive accumulation of neurotoxic factors will induce more glial cells in the nervous system, resulting in secondary damage to the nervous system and hindering the regeneration of neuronal cells. Therefore, neuritis is also regarded as the pathogenesis of a variety of neurodegenerative diseases, including brain injury, Parkinson’s disease (PD), and Alzheimer’s disease (AD) [2,3,4]. Due to the limited cell renewal and regeneration ability of nerve tissue, the CNS is extremely vulnerable in the uncontrolled process of autoimmunity and inflammation. On the one hand, under normal operating conditions, neuroinflammation participates in various life activities, including phagocytosis, steroid release, free radical reduction, and cell repair, showing affinity and anti-inflammatory activity. On the other hand, under the conditions of disease or external stimulation, neuroinflammation takes part in the excessive release of oxidative molecules and neurotoxic molecules, impairs the physiological function of normal neurons, and causes the failure of synaptic function, which is manifested as pro-inflammatory activity [5,6]. In general, uncontrolled or persistent neuroinflammation, whether as an inducer or as a cascade feedback response, may drive chronic and progressive neurodegenerative processes.

Lipopolysaccharide (LPS) is the main component of the cell wall of gram-negative bacteria, which is widely used to establish the model of neuroinflammation in vivo [7]. According to the literature, LPS induction could increase the release of proinflammatory factors, including NO, PGE2, TNF-α, IL-6, and IL-1β, and through the classical NF-κB and MAPK signaling pathway-mediated inflammatory response. NF-κB is a key nuclear transcription factor and the first responder to harmful cell stimulation, which regulates the expression of a variety of pro-inflammatory genes [5]. Many investigations have shown that inhibition of NF-κB pathway improves LPS-stimulated neuroinflammation in mice [8]. Additionally, MAPKs has been widely involved in the regulation of neuroinflammation [9]. The NLRP3 inflammasome is a protein complex in the innate immune system that recognizes pathogen- and risk-related molecular patterns. Its over-activation can release a large number of inflammatory cytokines, resulting in tissue damage and inflammatory conditions. Previous works have proved that LPS activates the NLRP3 inflammasome pathway and promotes the massive release of caspase-1, IL-1β, and IL-18 [10,11]. The NLRP3 inflammasome is implicated in the progression of neuroinflammation both in vitro and in vivo [12].

**Figure 1:**
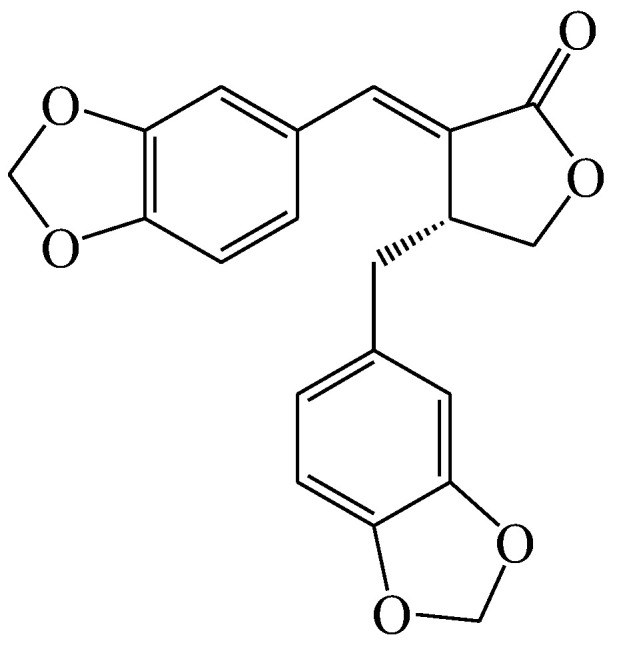
Chemical structure of savinin.

At present, there are still no effective methods and drugs for the treatment of neuroinflammation-related diseases. In recent years, the search for natural products with significant anti-neuritis activity from TCM has attracted more and more attention. Studies have demonstrated that many TCMs can prevent and treat neurological diseases with few adverse reactions [13,14]. *Eleutherococcus henryi* Oliv. is a well-known TCM, which is commonly used to treat mental fatigue and amnesia, as well as inflammatory conditions, including rheumatism, arthritis, and hepatitis [15,16] (The plant name has been checked with World Flora Online (www.worldfloraonline.org, accessed on 26 August 2022) and Medicinal Plant Names Services (http://mpns.kew.org, accessed on 26 August 2022). Savinin is a natural lignan isolated from the roots of *E. henryi* (Figure 1) [17]. A study has shown that savinin significantly reduced the production of TNF-α in LPS-induced RAW264.7 macrophages, and the proliferation of T cells treated with concanavalin (Con A) and mediated by the non-polar butyrolactone ring [18]. Also, it has exhibited the prominent inhibitory effects of the production of PGE2 in TPA-induced rat peritoneal macrophages [19]. In addition, it was reported that savinin demonstrated potent spermicidal, insecticidal, and anti-estrogenic activities [20,21]. Furthermore, recent studies have reported that savinin is one of several potential drug candidates being considered in the search for antibiotics against COVID-19, using the methods of network pharmacological analysis [22,23,24]. In our previous studies, treatment with savinin suppressed LPS-stimulated neuroinflammation in BV2 microglia [16,17]. Herein, the anti-neuroinflammatory effects of savinin and the possible underlying mechanisms were investigated in LPS-treated mice.

## 2. Results

### 2.1. Savinin Ameliorated LPS-Induced Histopathological Changes

**Figure 2:**
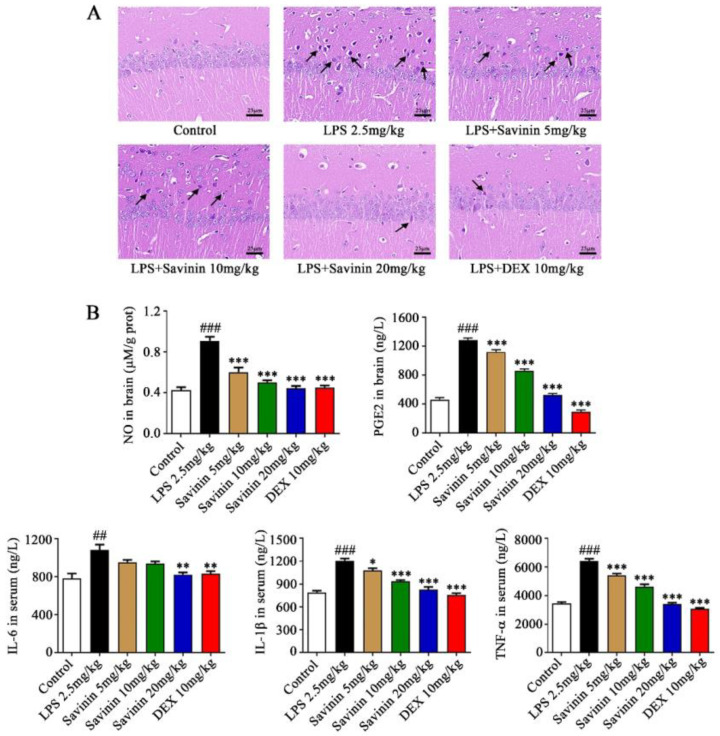
Savinin ameliorated histopathological changes and pro-inflammatory factor expressions in hippocampus and serum of neuroinflammation-affected mice. Groups of mice were administered savinin (5, 10, 20 mg/kg) and DEX (10 mg/kg) by gavage once daily for a continuous 7 days. On the 5th day of continuous pre-administration, LPS (2.5 mg/kg) was injected into the lateral ventricles of mice for modeling 48 h. On the 7th day, the mice were sacrificed and the serum and brain tissues (hippocampus) were collected for further analysis. (**A**) Images illustrating H&E staining of histopathological changes in brain tissue slices. Black arrows show neurons that were crinkled and deeply stained. Magnification: ×400. Scale bar, 25 μm. (**B**) NO and PGE2 levels in brain hippocampus, as well as IL-6, IL-1β, and TNF-α levels in serum were determined using ELISA. Data are shown as mean ± SD. ## *p* < 0.01, ### *p* < 0.001 vs. control, * *p* < 0.05, ** *p* < 0.01, *** *p* < 0.001 vs. LPS. *n* = 3.

HE staining assay was carried out to assess histopathological changes in the hippocampus of mice. HE staining results of brain slices indicated that the structure of brain tissues of mice in the control group was normal, the neurons in the hippocampus were arranged neatly and closely, and the neuron number was not reduced. At the same time, no obvious degeneration was found in the cells. By contrast, LPS treatment triggered an inflammatory reaction in brain tissue, as shown by the black arrow, and the structure of the brain tissue was abnormal. The arrangement of neurons in the hippocampus was loose and disordered, and the number of cells decreased remarkably. A large number of cell bodies were crinkled and deeply stained, and the peripheral spaces were significantly enlarged. Intriguingly, a dose-dependent improvement in histopathological changes induced by LPS was observed following pretreatment with savinin (5, 10, and 20 mg/kg) (Figure 2A).

### 2.2. The Effect of Savinin on Expression of Inflammatory Factors in LPS-Treated Mice

**Figure 3:**
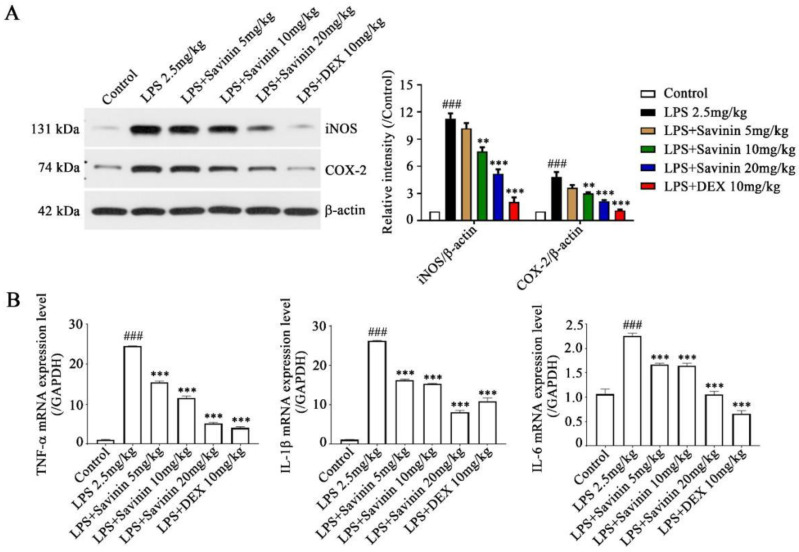
Savinin suppressed LPS-induced neuroinflammatory response in brain hippocampus of mice. Groups of mice were administered savinin (5, 10, 20 mg/kg) and DEX (10 mg/kg) by gavage once daily for a continuous 7 days. On the 5th day of continuous pre-administration, LPS (2.5 mg/kg) was injected into the lateral ventricles of mice for modeling 48 h. On the 7th day, the mice were sacrificed and the serum and brain tissues (hippocampus) were collected for further analysis. (**A**) The protein expression levels of iNOS and COX-2 were detected by Western blotting. (**B**) The mRNA expression of TNF-α, IL-1β, and IL-6 were determined using qRT-PCR. Data are shown as mean ± SD. ### *p* < 0.001 vs. control, ** *p* < 0.01, *** *p* < 0.001 vs. LPS. *n* = 3.

To verify that the effects of savinin on neuroinflammation is related to the inhibition of proinflammatory cytokine release, ELISA assay was executed to evaluate the levels of NO and PGE2 in brain hippocampus, as well as IL-6, IL-1β, and TNF-α in serum. Simultaneously, qRT-PCR assay was performed to determine the mRNA expression of TNF-α, IL-1β, and IL-6 in brain hippocampus. Our results exhibited that the production of NO, PGE2, TNF-α, IL-1β, and IL-6 dramatically increased in LPS-treated mice and this performance was offset following savinin pretreatment (Figure 2B and Figure 3B). Additionally, in order to measure the levels of iNOS and COX-2 expression in hippocampus, Western blotting assays were conducted. As shown in Figure 3A, pre-treatment with savinin markedly inhibited the expression of iNOS and COX-2 in mice treated with LPS. These results make clear that savinin suppressed the LPS-stimulated neuroinflammatory response.

### 2.3. The Effect of Savinin on MAPK/NF-κB Signaling Pathway

**Figure 4:**
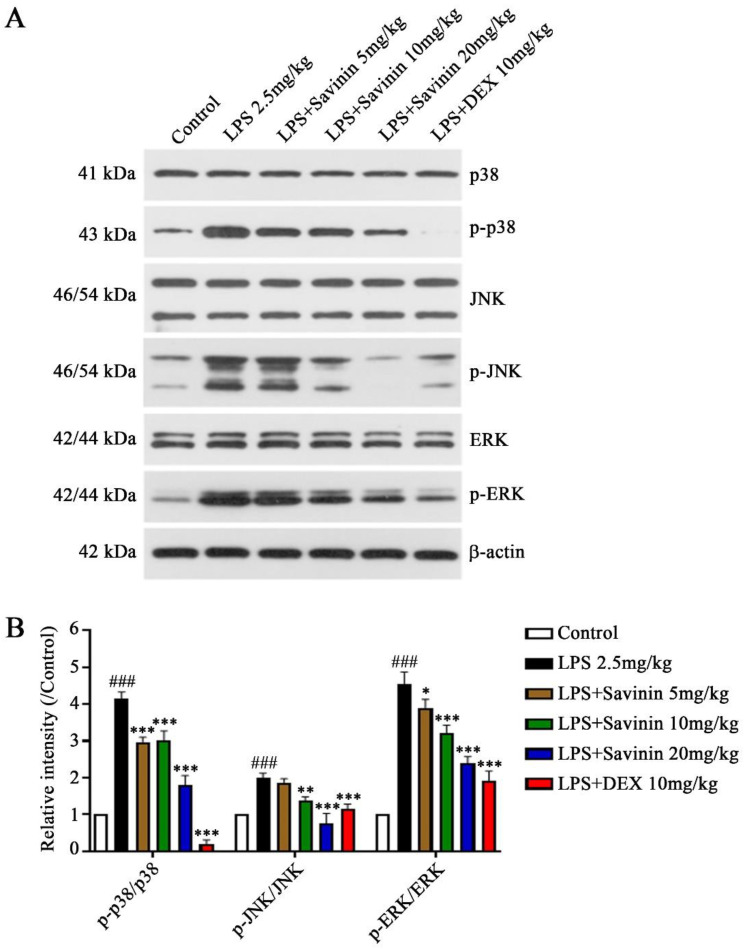
Savinin inhibited LPS-induced activation of MAPK signaling pathway in hippocampus. Groups of mice were administered savinin (5, 10, 20 mg/kg) and DEX (10 mg/kg) by gavage once daily for a continuous 7 days. On the 5th day of continuous pre-administration, LPS (2.5 mg/kg) was injected into the lateral ventricles of mice for modeling 48 h. On the 7th day, the mice were sacrificed and the serum and brain tissues (hippocampus) were collected for further analysis. (**A**) The protein expression levels of p38, p-p38, JNK, p-JNK, ERK, and p-ERK were detected by Western blotting. (**B**) Semi-quantification of p-p38, p-JNK, and p-ERK. Data are shown as mean ± SD. ### *p* < 0.001 vs. control, * *p* < 0.05, ** *p* < 0.01, *** *p* < 0.001 vs. LPS. *n* = 3.

**Figure 5:**
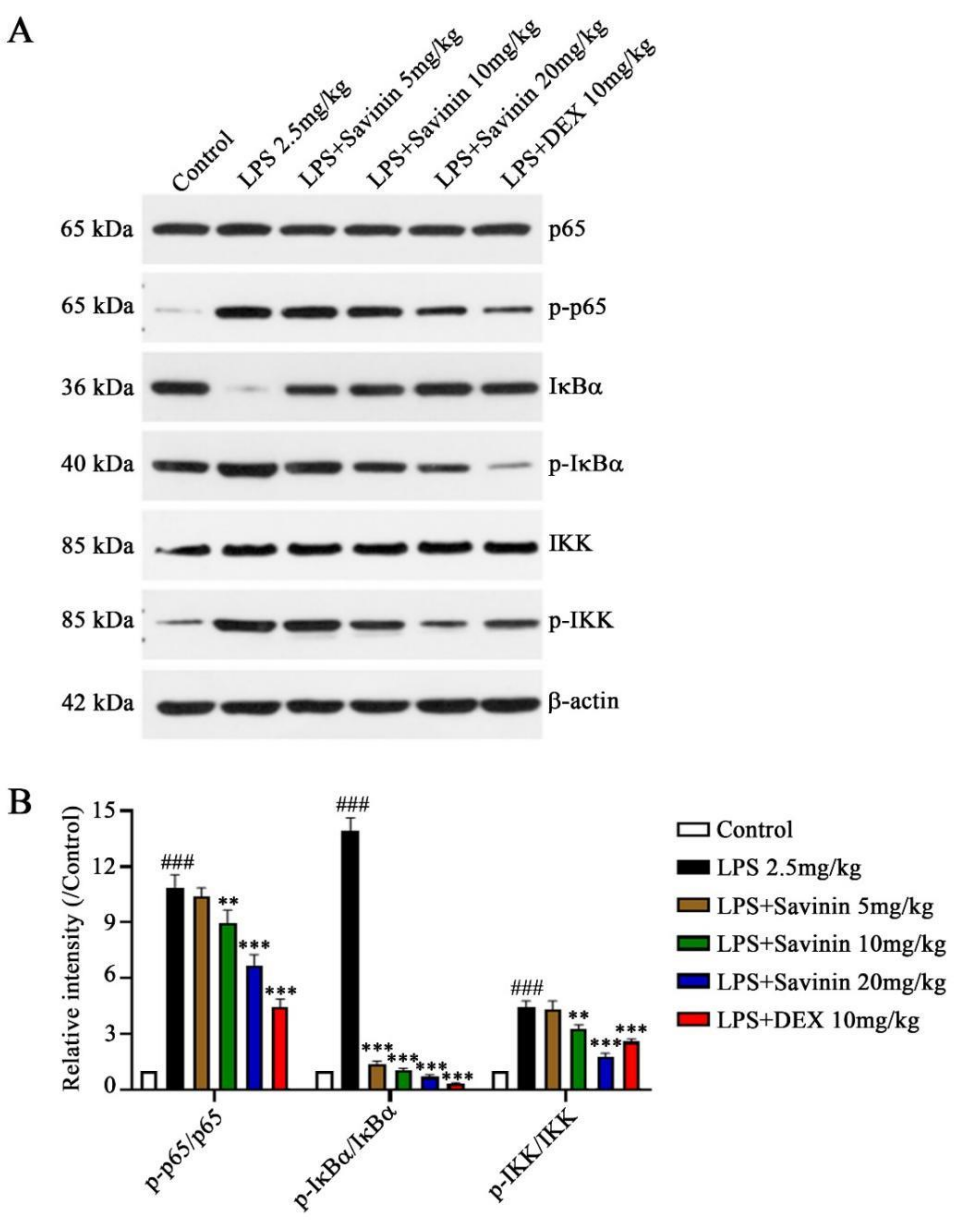
Savinin inhibited LPS-induced activation of NF-κB signaling pathway in hippocampus. Groups of mice were administered savinin (5, 10, 20 mg/kg) and DEX (10 mg/kg) by gavage once daily for continuous 7 days. On the 5th day of continuous pre-administration, LPS (2.5 mg/kg) was injected into the lateral ventricles of mice for modeling 48 h. On the 7th day, the mice were sacrificed and the serum and brain tissues (hippocampus) were collected for further analysis. (**A**) The protein expression levels of p65, p-p65, IκBα, p-IκBα, IKK, and p-IKK were detected by Western blotting. (**B**) Semi-quantification of p-p65, p-IκBα, and p-IKK. Data are shown as mean ± SD. ### *p* < 0.001 vs. control, ** *p* < 0.01, *** *p* < 0.001 vs. LPS. *n* = 3.

**Figure 6:**
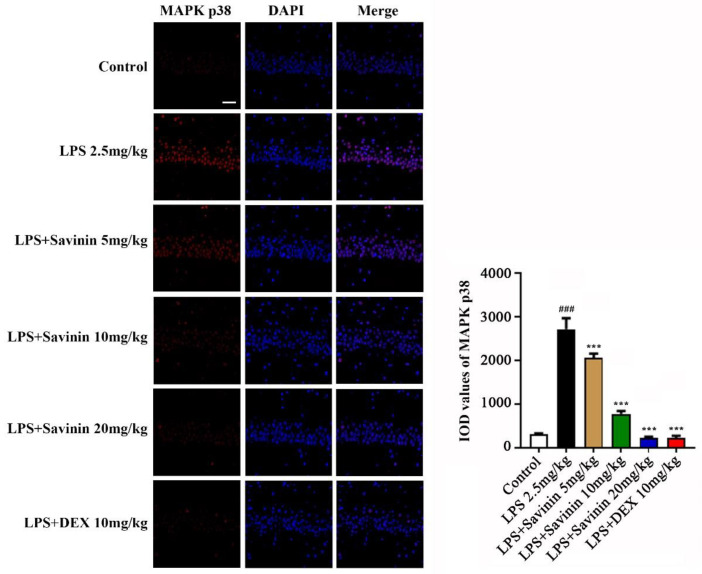
Savinin inhibited MAPK p38 expression induced by LPS in brain hippocampus (CA1) of mice. Groups of mice were administered savinin (5, 10, 20 mg/kg) and DEX (10 mg/kg) by gavage once daily for a continuous 7 days. On the 5th day of continuous pre-administration, LPS (2.5 mg/kg) was injected into the lateral ventricles of mice for modeling 48 h. On the 7th day, the mice were sacrificed and the serum and brain tissues (hippocampus) were collected for further analysis. Representative images of MAPK p38 (red) staining in brain tissues. Nuclei are stained with 4, 6-diaminido-2-phenylindole (DAPI, blue). Values are expressed as mean ± SD (*n* = 3). ### *p* < 0.001 vs. control, *** *p* < 0.001 vs. LPS. *n* = 3. Magnification: ×400. Scale bar, 25 μm.

**Figure 7:**
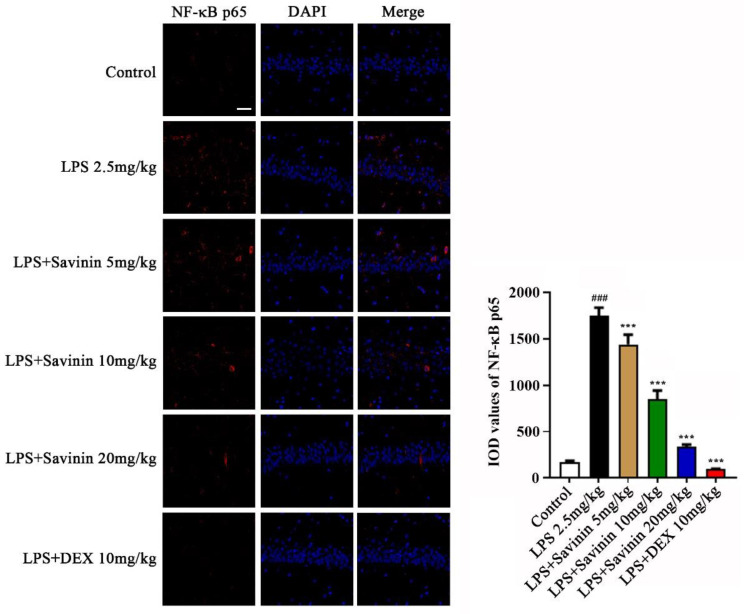
Savinin prevents NF-κB p65 nuclear translocation induced by LPS in brain hippocampus (CA1) of mice. Groups of mice were administered savinin (5, 10, 20 mg/kg) and DEX (10 mg/kg) by gavage once daily for a continuous 7 days. On the 5th day of continuous pre-administration, LPS (2.5 mg/kg) was injected into the lateral ventricles of mice for modeling 48 h. On the 7th day, the mice were sacrificed and the serum and brain tissues (hippocampus) were collected for further analysis. Representative images of NF-κB p65 (red) staining in brain tissues. Nuclei are stained with 4, 6-diaminido-2-phenylindole (DAPI, blue). Values are expressed as mean ± SD (*n* = 3). ### *p* < 0.001 vs. control, *** *p* < 0.001 vs. LPS. *n* = 3. Magnification: ×400. Scale bar, 25 μm.

We used Western blotting and immunofluorescence staining assays to detect the expression of MAPK/NF-κB signaling pathway-related proteins so as to clarify the mechanism of savinin inhibiting the release of inflammatory mediators. The finding indicated that savinin alleviated LPS-stimulated MAPK activation and inhibited the phosphorylation of p38, JNK, and ERK caused by LPS (Figure 4). Meanwhile, it down-regulated the expression levels on p-p65, p-IκBα, and p-IKK, as well as the degradation of IκBα (Figure 5). Furthermore, the immunofluorescence staining results demonstrated that LPS treatment up-regulated the expression of MAPK p38 and NF-κB p65 in the hippocampus (CA1); however, savinin pretreatment counteracted these effects (Figure 6 and Figure 7). These investigations reveal that savinin decreased the LPS-triggered activation of MAPK and NF-κB signaling pathways.

### 2.4. The Effect of Savinin on Formation of the NLRP3 Inflammasome Complex

**Figure 8:**
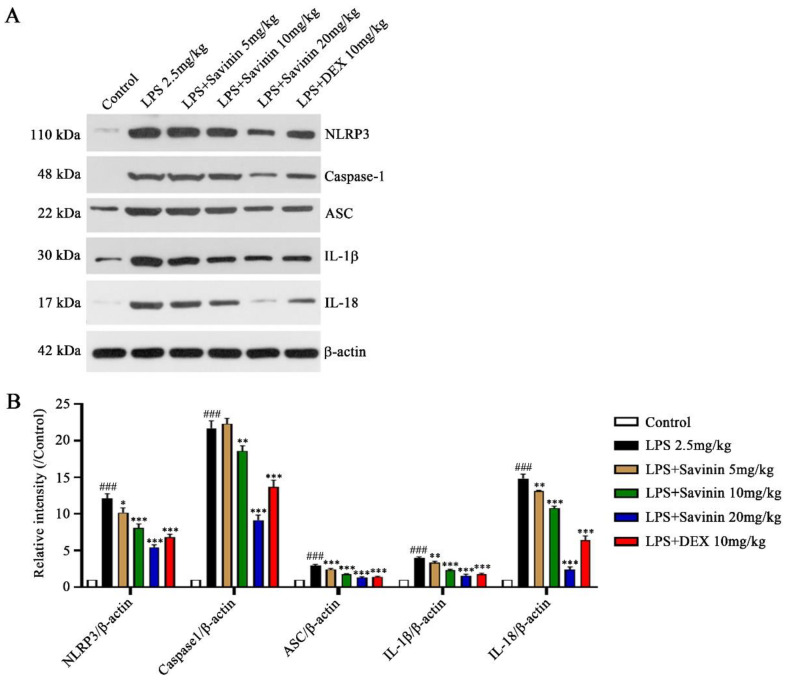
Savinin inhibited LPS-induced activation of NLRP3 signaling pathway in hippocampus. Groups of mice were administered savinin (5, 10, 20 mg/kg) and DEX (10 mg/kg) by gavage once daily for a continuous 7 days. On the 5th day of continuous pre-administration, LPS (2.5 mg/kg) was injected into the lateral ventricles of mice for modeling 48 h. On the 7th day, the mice were sacrificed and the serum and brain tissues (hippocampus) were collected for further analysis. (**A**) The protein expression levels of NLRP3, caspase-1, ASC, IL-1β, and IL-18 were detected by Western blotting. (**B**) Semi-quantification of NLRP3, caspase-1, ASC, IL-1β, and IL-18. Data are shown as mean ± SD. ### *p* < 0.001 vs. control, * *p* < 0.05, ** *p* < 0.01, *** *p* < 0.001 vs. LPS. *n* = 3.

**Figure 9:**
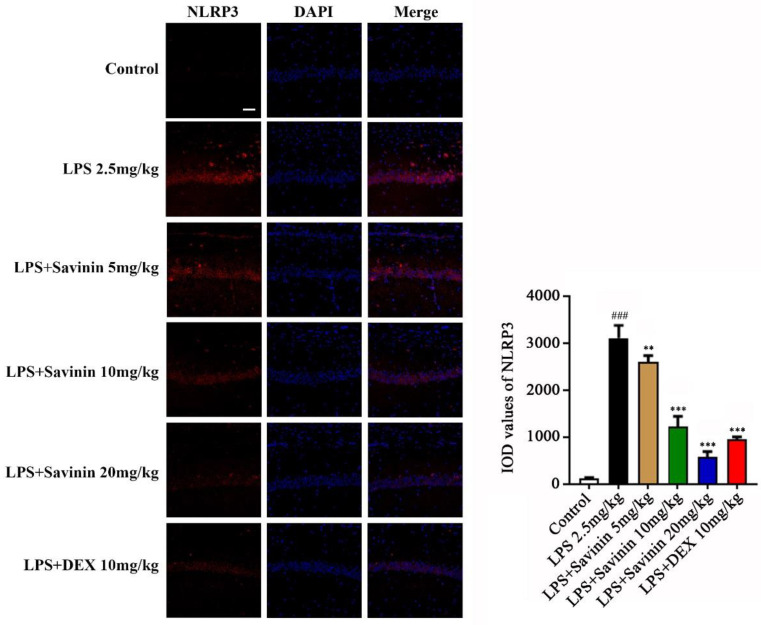
Savinin inhibited NLRP3 expression induced by LPS in brain hippocampus (CA1) of mice. Groups of mice were administered savinin (5, 10, 20 mg/kg) and DEX (10 mg/kg) by gavage once daily for a continuous 7 days. On the 5th day of continuous pre-administration, LPS (2.5 mg/kg) was injected into the lateral ventricles of mice for modeling 48 h. On the 7th day, the mice were sacrificed and the serum and brain tissues (hippocampus) were collected for further analysis. Representative images of NLRP3 (red) staining in brain tissues. Nuclei are stained with 4, 6-diaminido-2-phenylindole (DAPI, blue). Values are expressed as mean ± SD (*n* = 3). ### *p* < 0.001 vs. control, ** *p* < 0.01, *** *p* < 0.001 vs. LPS. *n* = 3. Magnification: ×400. Scale bar, 25 μm.

Western blot and immunofluorescence staining techniques were used to examine the expression of proteins associated with the NLRP3 inflammasome pathway in brain hippocampus for the purpose of exploring the effect of savinin on NLRP3 inflammasome activation. Treatment with LPS increased the protein expression levels of NLRP3, caspase-1, ASC, IL-1β, and IL-18 in brain. However, savinin pretreatment reversed these changes (Figure 8). Moreover, the immunofluorescence results demonstrated that LPS treatment enhanced NLRP3 expression, and this effect was weakened by savinin pretreatment (Figure 9).

### 2.5. Molecular Docking Analysis

**Figure 10:**
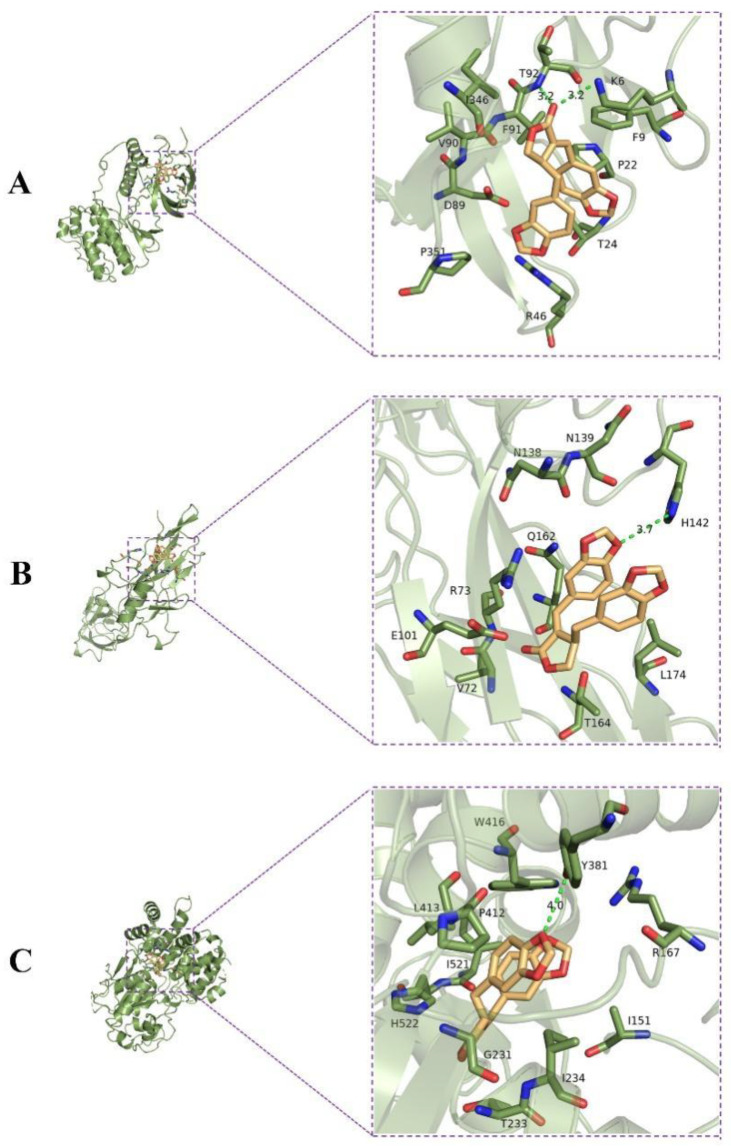
Molecular docking experiment showing the binding of savinin (yellow) with MAPK13 (**A**), NF-κB (**B**), and NLRP3 (**C**) proteins (light-green) as analyzed using Pymol software.

The results of molecular docking analysis showed that small-molecule ligand savinin bound well to the active site of receptor proteins MAPK, NF-κB, and NLRP3 with well-fitting mode. All active sites had a binding energy below −6 kcal/mol, which revealed that the binding between ligand and receptors was spontaneous (Table 1). Savinin bound to MAPK13 by forming hydrogen bonds with amino acid residues T92 (Thr92) and K6 (Lys6), hydrophobic bonds with amino acid residues F9 (Phe9), P22 (Pro22), T24 (Thr24), R46 (Arg46), P351 (Pro351), D89 (Asp89), V90 (Val90), F91 (Phe91), and I346 (Ile346), with a binding energy of −7.1 kcal/mol (Figure 10A). Meanwhile, an analysis of the binding energy between savinin and NF-κB revealed that it was −6.7 kcal/mol. Savinin formed a hydrogen bond with H142 (His142) and hydrophobic bonds with L174 (Leu174), T164 (Thr164), V72 (Val72), E101 (Glu101), R73 (Arg73), Q162 (Gln162), N138 (Asn138), and N139 (Asn139) (Figure 10B). Moreover, by forming a hydrogen bond with Y381 (Tyr381) and hydrophobic bonds with R167 (Arg167), I151 (Ile151), I234 (Ile234), T233 (Thr233), G231 (Gly231), H522 (His522), I521 (Ile521), P412 (Pro412), L413 (Leu413), and W416 (Trp416), savinin bound well to NLRP3, with a binding energy of −9.1 kcal/mol (Figure 10C).

## 3. Discussion

The development process of neurodegenerative diseases is closely related to the activation of microglia, the release of inflammatory mediators, and the damage of neurons. Neuroinflammation provoked by bacteria and viruses, as well as other biologic pathogens, is a common risk factor for neurodegenerative diseases [25]. To date, there is still a lack of effective strategies for the treatment of neurodegenerative diseases. Hence, new candidates and drugs that have better clinical therapeutic effects for treating neurodegenerative diseases are urgently needed. Located between the thalamus and the medial temporal lobe of the brain, the hippocampus is part of the limbic system and is mainly responsible for the storage, conversion, and orientation of short-term memory, as well as learning and emotions [26]. Many studies have shown that the hippocampus is involved in the pathological process of neurodegenerative diseases [27]. LPS, as the endotoxin from the cytoderm of gram-negative bacteria, is widely accepted and used for modeling of cerebral inflammation [7,8,9]. In this investigation, we evaluate the neuroprotective effects of savinin on an LPS-induced cerebral inflammation model.

The activated microglia release proinflammatory cytokines and signaling molecules, such as NO, PGE2, TNF-α, IL-1β, and IL-6, which aggravate the inflammatory response and accelerate the spread of neuroinflammation [5,8,28]. As our results show, savinin can delay the process of neuroinflammation by inhibiting the expression of synthase iNOS and COX-2, as well as the production and release of NO, PGE2, TNF-α, IL-1β, and IL-6. MAPKs and NF-κB signaling molecules are considered to be the main regulators of neuroinflammation in brain diseases. They transcribe and activate the above enzymes and proinflammatory cytokines to induce neuroinflammation [9]. In this study, we found that savinin mainly regulates MAPKs and NF-κB signaling pathways to inhibit the production and release of proinflammatory mediators, showing anti-neuroinflammatory activities.

Additionally, the NLRP3 inflammasome pathway is modulated by MAPK and NF-κB pathways [29]. NLRP3 is the most widely studied target of various types of inflammasomes that have been found so far, and has an effect on the brain [30]. The caspase-1 and ASC are activated by the NLRP3 inflammasome, which successively cleaves pro-IL-1β/18 into mature IL-1β/18 [31]. IL-1β/18 are the products of NLRP3 inflammasome activation and play an important role in the occurrence and development of neuroinflammation. Many types of neurological diseases are related to neuroinflammation mediated by the NLRP3 inflammasome, such as AD, PD, amyotrophic lateral sclerosis (ALS), multiple sclerosis (MS), and depression [32]. Therefore, finding the active substances that can inhibit the expression of NLRP3 from TCM will be an effective way to treat CNS diseases mediated by neuroinflammation. For instance, the aqueous extract of *Epimedii Folium* and *Curculiginis Rhizoma* had anti-neuroinflammatory effects on AD by suppressing the NLRP3 inflammasome activation and MAPK/NF-κB pathway [33,34]. *Artemisiae Iwayomogii* Herba inhibits LPS-induced nerve inflammation by regulating NF-κB/MAPK and NLRP3 signaling pathways [28]. Baicalin alleviates cognitive impairment and protects neurons from microglia-mediated neuroinflammation via inhibiting NLRP3 inflammasomes and the TLR4/NF-κB signaling pathway [12]. Nootkatone, a natural product isolated from *Alpiniae Oxyphyllae Fructus*, suppressed neuroinflammatory responses via modulating the NF-κB and NLRP3 pathways, leading to the remission of learning and memory impairment in an LPS-triggered mouse model of AD [35]. The natural polysaccharides (SCP2-1) obtained from *Schisandra chinensis* (Turcz.) Baill possess neuroprotective and cognition-improving activities in LPS-induced AD-like models by inhibiting the over-activation of the NLRP3 inflammasome, MAPK, and NF-κB pathways [36]. Similarly, our study demonstrated that savinin had anti-neuritis effects via inhibiting MAPK and NF-κB, as well as NLRP3 pathways in LPS-induced mice, suggesting that savinin may contribute to the treatment of neurodegenerative disorders. Although previous studies have proven that savinin has good inhibitory effects on other inflammatory diseases, it has also been confirmed that savinin has significant anti-neuroinflammatory activity in vitro [16,17,18,19]. This study further proved the anti-neuroinflammatory effects of savinin at the in vivo level for the first time, that is, savinin inhibits brain inflammation, and these effects are related to both MAPK/NF-κB and NLRP3 pathways.

Molecular docking is a method of designing drugs based on receptor characteristics and receptor–drug interactions. In this method, molecules (for instance, receptors and ligands) are simulated to determine their binding mode and affinity [37]. Computer-aided drug research has become increasingly dependent on molecular docking in recent years [38]. We carried out molecular docking analysis in the molecular operating environment to verify the results in vivo. Based on the results, savinin binds to MAPK, NF-κB, and NLRP3 active sites with a minimum binding energy under -6 kcal/mol, indicating that this is a spontaneous process. Our investigation results successfully verified the experimental objectives of molecular interaction.

This work provides new evidence for savinin to mitigate LPS-induced neuroinflammation in vivo. Overall, as a result of savinin treatment, LPS-stimulated neurogenic inflammation and the activation of MAPK, NF-κB, and the NLRP3 inflammasomes in brain tissue were reduced. Nevertheless, a more detailed analysis of the signal pathways affected by savinin on MAPK/NF-κB and NLRP3 is required. Gene-editing technology will support this work in subsequent research.

## 4. Materials and Methods

### 4.1. Reagents

Savinin (CAS no. 493-95-8, purity ≥ 98%) was isolated from the roots of *E. henryi*, as described previously [17]. Sigma Aldrich (St.Louis, MO, USA) provided LPS (cat. no. L-6529) and dexamethasone (DEX) (cat. no. HY-14648) for this study. Antibodies against iNOS (cat no. 13120), COX-2 (cat no. 12282), p38 (cat. no. 54470), p-p38 (cat. no. 4511), JNK (cat. no. 9252), p-JNK (cat. no. 9255), ERK (cat. no. 4695), p-ERK (cat. no. 4370), p65 (cat. no. 8242), p-p65 (cat. no. 13346), IκBα (cat. no. 4812), p-IκBα (cat. no. 2859), IKK (cat. no. 2682), p-IKK (cat. no. 2697), NLRP3 (cat. no. 15101), caspase 1 (cat. no. 24232), ASC (cat. no. 67824), IL-1β (cat. no. 31202), and IL-18 (cat. no. 57058) were purchased from CST Co. (Danvers, MA, USA); β-actin (cat. no. Ab8226) was purchased from Sigma Aldrich (St.Louis, MO, USA); TNF-α, IL-6, IL-1β, and GAPDH PCR primers were created by Shanghai shenggong Biological Engineering Co., Ltd. (Shanghai, China).

### 4.2. Experiments on Animals

Six-week-old male C57BL/6 mice were obtained from Hunan SiLaiKe Jingda Experimental Animal Center (Hunan, China). All mice were kept in a constant temperature and ventilation environment at 25 ± 1 °C in a light/dark cycle of 12 h with free access to food and water. After acclimatization for 7 days, 6 groups of mice were randomly divided: control group, LPS (2.5 mg/kg) group, LPS + savinin (5 mg/kg) group, LPS + savinin (10 mg/kg) group, LPS + savinin (20 mg/kg) group, and LPS + DEX (10 mg/kg) group (*n* = 6/group). We administered savinin and DEX intragastrically to the savinin and DEX groups by gavage for 7 days, as well as physiological saline continuously to the control and LPS groups. On the 5th day of continuous pre-administration, mice in the control group were injected with an equal volume of physiological saline into the lateral ventricles, mice in other groups were injected with 2.5 mg/kg LPS into the lateral ventricles for modeling 48 h. On the 7th day, mice were anesthetized with 10% chloral hydrate, and the serum and hippocampus of the brain were collected and stored at −80 ℃ for further analysis. All experiments were performed in accordance with the guidelines of the Institutional Animal Care and Use Committee of China and were approved by the Ethical Committee of Gannan Medical University (Approval Number: GMU202020).

### 4.3. Hematoxylin-Eosin (HE) Staining

Pieces of the mice’s brain tissue, including the hippocampus, were fixed in 4% paraformaldehyde and embedded in paraffin, then sections were cut into 5 mm and dewaxed. After staining with hematoxylin and eosin (HE) solution, respectively, the specimens were dehydrated and covered with coverslips. In the end, a microscope (NIKON DS-U3, Nikon, Tokyo, Japan) was used to examine the stained specimens.

### 4.4. Nitrite (NO) and Prostaglandin E2 (PGE2) Determination

In order to assess the levels of nitric oxide (NO) and prostaglandin E2 (PGE2) in hippocampal brain homogenates, a standard commercial kit (Elabscience Biotechnology Co., Ltd., Wuhan, China) and an ELISA kit (Elabscience biotechnology Co., Ltd., Wuhan, China) were used, respectively.

### 4.5. Enzyme-Linked Immunosorbent Assay (ELISA)

Serum levels of TNF-α, IL-1β, and IL-6 were measured using standard ELISA kits (Elabscience biotechnology Co., Ltd., Wuhan, China) following the manufacturer’s instructions.

### 4.6. Quantitative Real-Time Polymerase Chain Reaction (qRT-PCR)

Total RNA content of the hippocampi of the mice’s brain tissue was extracted using Trizol reagent according to the manufacturer’s instructions (ER501-01, TRANS, Beijing, China). Reverse transcription of cDNA was conducted using EasyScript^®^ All-in-One First-Strand cDNA Synthesis SuperMix for qPCR kit (AE341, TRANS, Beijing, China). The qPCR was executed by using PerfectStart^®^ Green qPCR SuperMix (AQ601, TRANS, Beijing, China). The 2^−ΔΔCT^ method was used to measure relative mRNA expression based on GAPDH as a reference gene. A list of primers for the genes is provided in Table 2.

### 4.7. Western Blotting

Proteins were extracted from the hippocampi of the brain tissue of the mice using RIPA lysis buffer containing protease and phosphatase inhibitors. The lysate was centrifuged at 14,000 rpm/10 min at 4 °C and the concentration of proteins was quantified by the BCA method. Ten percent SDS-PAGE gels were used to separate each sample’s proteins, which were then transferred to PVDF membranes. All membranes were blocked with 5% skim milk for 1 h at room temperature, then incubated at 4 °C overnight with respective primary antibodies against iNOS (1:1000), COX-2 (1:1000), p38 (1:1000), p-p38 (1:1000), JNK (1:1000), p-JNK (1:1000), ERK (1:1000), p-ERK (1:1000), p65 (1:1000), p-p65 (1:800), IκBα (1:1000), p-IκBα (1:1000), IKK (1:1000), p-IKK (1:1000), NLRP3 (1:1000), caspase 1 (1:1000), ASC (1:1000), IL-1β (1:1000), IL-18 (1:1000), and β-actin (1:2000). The membranes were sequentially incubated with the secondary antibodies (1:5000) for 1 h at room temperature. The protein bands were developed using enhanced chemiluminescence reagent (ECL, Thermo Scientific, Waltham, MA, USA), and ImageJ software was used for semi-quantification.

### 4.8. Immunofluorescence (IF) Staining

Sections from hippocampal cornu ammonis (CA1) of mice brain tissue fixed in 4% paraformaldehyde were dewaxed and hydrated, then incubated with 3% hydrogen peroxide at room temperature for 10 min. Sections were permeabilized and blocked for 30 min with 5% BSA in PBS containing 0.3% Triton X-100. All brain sections were incubated with the primary antibodies against MAPK p38, NF-κB p65, and NLRP3 at 4 °C overnight. After 1 day, they were incubated with the secondary antibody, Alexa 488. Fluorescence was visualized with an inverted fluorescent microscope (LSM880, Zeiss, Oberkochen, Germany).

### 4.9. Molecular Docking

Schrodinger software was used for docking of savinin and protein molecules. The structure of savinin was drawn and converted to PDB file format by using Schrodinger software. Crystal structure files in the PDB format of MAPK (PDB ID: 4EVJ), NF-kB (PDB ID: 2I9T), and NLRP3 (PDB ID: 7ALV) were obtained from the PDB website (http://www.rcsb.org, accessed on 30 August 2022). Savinin and protein molecules were added and optimized into Schrodinger software, then water molecules were removed, atomic types were set, the receptor grid was run, and the final molecule files were saved as PDB files. A visualization of the binding between the compound and proteins was performed with Pymol 2.6 (Schrodinger, New York, NY, USA). 

### 4.10. Statistical Analysis

Statistical data are presented as means and standard deviations (SD) based on at least three independent experiments. To compare three or more groups, an analysis of variance (ANOVA) was used, followed by a Tukey’s multiple comparison test. Statistical analysis was performed using GraphPad Prism software, version 3.03 (GraphPad Software Inc., San Diego, CA, USA).

## 5. Conclusions

Taken together, our work indicates that savinin ameliorates LPS-triggered neuroinflammation in vivo by modulating MAPK/NF-κB signaling pathway and NLRP3 inflammasome activation. For that reason, savinin may be a useful anti-neuroinflammatory candidate, which may have therapeutic potential for neuroinflammation-related CNS disorders.

## Figures and Tables

**Table 1:** Binding affinities and binding residues, as determined by molecular docking.

Receptors	Binding Affinity (kcal/mol)	Binding Residues
MAPK13	−7.1	T92 (Thr92), K6 (Lys6), F9 (Phe9), P22 (Pro22), T24 (Thr24), R46 (Arg46), P351 (Pro351), D89 (Asp89), V90 (Val90), F91 (Phe91), I346 (Ile346)
NF-κB	−6.7	H142 (His142), L174 (Leu174), T164 (Thr164), V72 (Val72), E101 (Glu101), R73 (Arg73), Q162 (Gln162), N138 (Asn138), N139 (Asn139)
NLRP3	−9.1	Y381 (Tyr381), R167 (Arg167), I151 (Ile151), I234 (Ile234), T233 (Thr233), G231 (Gly231), H522 (His522), I521 (Ile521), P412 (Pro412), L413 (Leu413), W416 (Trp416)

**Table 2:** Primer sequences for quantitative real-time PCR.

Gene	Sequence (5’-3’)
GAPDH	Forward: TCAACGGCACAGTCAAGG Reverse: TGAGCCCTTCCACGATG
TNF-α	Forward: ATCCGCGACGTGGAACTG Reverse: ACCGCCTGGAGTTCTGGAA
IL-1β	Forward: GAGCACCTTCTmCCTTCATCTT Reverse: TCACACACCAGCAGGTT
IL-6	Forward: TCCGGAGAGGAGACTTCACA Reverse: TTGCCATTGCACAACTCTTTTC

## Data Availability

The data that support the findings of this study are available from the corresponding author upon reasonable request.

## Sample Availability

Samples of the compounds are available from the authors.

## Abbreviations

LPS, Lipopolysaccharide; TCM, traditional Chinese medicine; CNS, central nervous system; NO, nitric oxide; PGE2, prostaglandin E2; iNOS, inducible nitric oxide synthase; COX-2, cyclooxygenase-2; IL-1β, interleukin-1β; IL-6, interleukin-6; TNF-α, tumor necrosis factor-α; MAPKs, mitogen-activated protein kinases; JNK, c-Jun N-terminal kinase; p-JNK, phosphorylated JNK; ERK, extracellular signal-regulated kinase; p-ERK, phosphorylated ERK; NF-κB, nuclear-factor-kappa B; IκBα, inhibitor of NF-κB; p-IκBα, phosphorylated IκBα; IKK, IκB kinase; p-IKK, phosphorylated IκB kinase; NLRP3, nucleotide-binding domain-like receptor protein 3; ASC, apoptosis-associated speck-like protein; IL-18, interleukin-18; DAPI, 4′,6-diamidino-2-phenylindole; ELISA, enzyme-linked immunosorbent assay; qRT-PCR, quantitative real-time polymerase chain reaction; IF, immunofluorescence; SD, standard deviation; ANOVA, analysis of variance; AD, Alzheimer’s disease; PD, Parkinson’s disease; ALS, amyotrophic lateral sclerosis; MS, multiple sclerosis; DEX, dexamethasone.
